# “Cre/loxP plus BAC”: a strategy for direct cloning of large DNA fragment and its applications in *Photorhabdus luminescens* and *Agrobacterium tumefaciens*

**DOI:** 10.1038/srep29087

**Published:** 2016-07-01

**Authors:** Shengbiao Hu, Zhengqiang Liu, Xu Zhang, Guoyong Zhang, Yali Xie, Xuezhi Ding, Xiangtao Mo, A. Francis Stewart, Jun Fu, Youming Zhang, Liqiu Xia

**Affiliations:** 1 Hunan Provincial Key Laboratory of Microbial Molecular Biology-State Key Laboratory Breeding Base of Microbial Molecular Biology, College of Life Science, Hunan Normal University, Changsha, 410081, People’s Republic of China; 2 Shandong University-Helmholtz Institute of Biotechnology, State Key Laboratory of Microbial Technology, School of Life Science, Shandong University, Shanda Nanlu 27, Jinan, 250100, People’s Republic of China; 3 Department of Genomics, Dresden University of Technology, BioInnovations-Zentrum, Tatzberg 47-51, Dresden, 01307, Germany

## Abstract

Heterologous expression has been proven to be a valid strategy for elucidating the natural products produced by gene clusters uncovered by genome sequencing projects. Efforts have been made to efficiently clone gene clusters directly from genomic DNA and several approaches have been developed. Here, we present an alternative strategy based on the site-specific recombinase system Cre/loxP for direct cloning gene clusters. A type three secretion system (T3SS) gene cluster (~32 kb) from *Photorhabdus luminescens* TT01 and DNA fragment (~78 kb) containing the siderophore biosynthetic gene cluster from *Agrobacterium tumefaciens* C58 have been successfully cloned into pBeloBAC11 with “Cre/loxP plus BAC” strategy. Based on the fact that Cre/loxP system has successfully used for genomic engineering in a wide range of organisms, we believe that this strategy could be widely used for direct cloning of large DNA fragment.

Owing to the rapidly evolving development in next-generation sequencing technologies, the number of deposited genome sequences has grown exponentially in recent years^1^. Genomic analysis of sequenced microorganisms has resulted in the discovery of a remarkably large number of orphan biosynthetic gene clusters, which are potentially a treasure chest of novel bioactive compounds with pharmaceutical applications^2^,^3^. Recently, efforts have been committed to opening this treasure chest and several strategies for awakening silent gene clusters have been developed^4^,^5^. These strategies include the whole pathway heterologous expression, which has the advantage that if a cluster is introduced into a host organism, and a new metabolite appears, one can ascribe it with confidence to the cluster^6^,^7^.

Cloning the complete biosynthetic gene clusters which can range in size up to 100 kb is among the first, and in some cases the most difficult procedures in the whole pathway heterologous expression strategy. The traditional cosmid/fosmid approach which contains time-consuming and labor-intensive library construction and screening steps can hardly meet the requirement for high-throughput discovery of new natural products^8^. Recently, several approaches have been developed to facilitate more efficient cloning of targeted gene clusters directly from genomic DNA, including *oriT*-directed cloning, phage φBT1 integrase-mediated site-specific recombination, transformation-associated recombination (TAR) and RecET-mediated linear-plus-linear homologous recombination (LLHR). The *oriT*-directed cloning developed dedicatedly for genetically tractable Gram-negative bacteria has been well validated in *Burkholderia pseudomallei* and *Sinorhizobium meliloti*^9^,^10^. The phage φBT1 integrase-mediated site-specific recombination has been used to successfully clone three antibiotic biosynthetic gene clusters from *Streptomyces* species^11^. The utility of TAR cloning which takes advantage of the natural *in vivo* homologous recombination of *Saccharomyces cerevisiae* was exemplified by its applications to capturing enterocin gene cluster from *Salinispora pacifica*, taromycin A biosynthetic gene cluster from *Saccharomonospora* sp., as well as large biosynthetic gene clusters from environmental DNA samples^12^,^13^,^14^,^15^,^16^,^17^. Ten megasynthetase gene clusters (each 10 ~ 52 kb in length) have been successfully directly cloned from the genome of *Photorhabdus luminescens* by using RecET-mediated LLHR^18^.

Since its emergence, RecET-mediated LLHR has been extensively applied to heterologous expression of secondary metabolite gene clusters from various microorganisms, such as sevadicin gene cluster from *Paenibacillus larvae*^19^, syringolin biosynthetic gene cluster from *Pseudomonas syringae*^20^, the glidobactin gene cluster from *Burkholderia* DSM7029^21^, and colibactin gene cluster from *E. coli* Nissle 1917^22^. However, in our attempt to heterologous expression of antitumor compound salinomycin, we failed to direct cloning the intact gene cluster (106 kb) from the genome of *Streptomyces albus* DSM41398 into pBeloBAC11 by using RecET-mediated LLHR. In order to circumvent the inefficiency of RecET-mediated LLHR in cloning large DNA fragment, two alternative strategies have been proposed and performed in parallel in our lab. In the first strategy, we separately cloned three fragments of salinomycin gene cluster using LLHR and then assembled them into an intact gene cluster by recombineering. Finally, we successfully reconstituted the salinomycin gene cluster and expressed it in the heterologous host *Streptomyces coelicolor* A3(2)^23^. Meanwhile, our second strategy which is based on the site-specific recombinase (SSR) system Cre/loxP has also been successfully developed to clone large DNA gene clusters from bacteria.

Cre recombinase, as well as Flp and Dre, belongs to the tyrosine recombinase family^24^,^25^. Cre recombinase catalyzes recombination between two specific 34-bp sites called loxP, with high specificity and efficiency. Cre/loxP system has long been recognized as the best SSR system for genome engineering, as it is effective not only in bacteria but also in eukaryotic cells. Depending on the location and orientation of the loxP sites, the Cre-mediated recombination between the loxP sites can lead to a specific deletion, translocation between chromosomes, or inversion of a DNA segment. If two loxP sites lie in the same orientation in the same DNA strand, Cre-mediated recombination will result in excision of the intervening DNA sequence and producing a circular DNA molecule^26^. It is therefore presumable that when a DNA replication origin is included the circularized intervening DNA could replicate as a plasmid in the corresponding organism(s). Based on this concept, we developed the “Cre/loxP plus BAC” strategy and validated it by direct cloning of gene clusters from *P. luminescens* strain TT01 (hereafter TT01) and *Agrobacterium tumefaciens* strain C58 (hereafter C58).

## Results

### Scheme for cloning large DNA fragment from bacterial genome by use of the “Cre/loxP plus BAC” strategy

**Figure 1:**
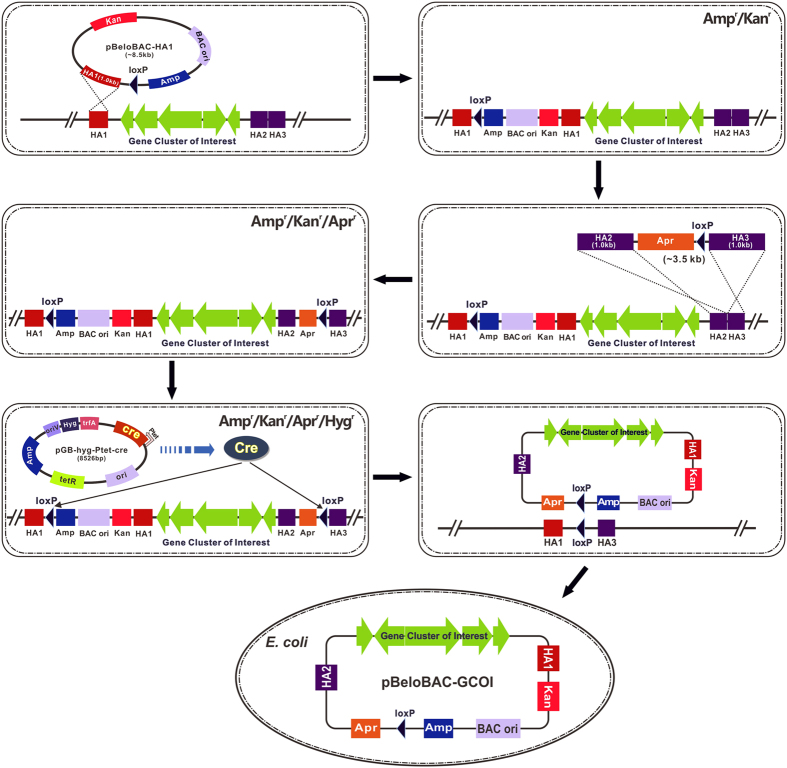
Schematic diagram of “Cre/loxP plus BAC” strategy for direct cloning large DNA fragment from bacterial chromosome. Briefly, integrate a loxP site and BAC backbone at the 5′ end of the gene cluster of interest via a single-crossover event. Integrate another loxP site at the 3′ end of the gene cluster of interest via a double-crossover event. Electroporate the Cre recombinase expression plasmid into bacterium and induce the expression of Cre recombinase. Isolate plasmid DNA from induced culture and electroporate into *E. coli*. Thus far, the complete gene cluster can be successfully cloned into pBeloBAC11. Amp, ampicillin resistance gene; Kan, kanamycin resistance gene; Apr, apramycin resistance gene; Hyg, hygromycin resistance gene.

Based upon the well-recognized fact that Cre recombinase could mediate recombination between two directly repeated loxP sites and consequently produce a circular DNA molecule containing the intervening DNA and a linear product containing the flanking DNA segments, it is presumable that when a DNA replication origin is included the excised intervening DNA could replicate as a plasmid in the corresponding organism(s). Therefore, we wonder whether Cre/loxP system could be adopted for direct cloning of complete gene clusters from bacterial genomes of which whole genome have been sequenced. Meanwhile, considering the large size of gene clusters, a BAC backbone could be suitable for stably maintaining larger DNA fragments in *E. coli*, a host which is readily amenable to genetic manipulation. The “Cre/loxP plus BAC” strategy is illustrated in Fig. 1.

Firstly, a pBeloBAC11-dervied vector, pBeloBAC-HA1 (Fig. S1A) harboring a loxP site and homology arm for chromosomal integration was constructed. As kanamycin exhibits broad-spectrum activity against bacterial species, *aph(3)II* gene which provides kanamycin resistance in bacteria was used to replace the chloramphenicol resistance gene resident in pBeloBAC11. Then, a loxP site which was incorporated into the DNA oligo was recombined into pBeloBAC11 along with ampicillin resistance gene. There exist EcoRІ, SacІ, BamHІ and HindIII unique restriction sites between kanamycin resistance gene and loxP site. A ~1 kb homology arm corresponding to the 5′ end of the gene clusters of interest (HA1) can be inserted into these unique restriction sites either by conventional digestion and ligation or by recombineering. The resultant vector pBeloBAC-HA1 is then electroporated into bacterium and integrated into the chromosome by a single-crossover event. Now, the first loxP site and BAC backbone have been successfully introduced at the 5′ end of the gene cluster of interest.

In order to introduce the second loxP site into bacterial chromosome and locate at the 3′ end of the gene clusters of interest, plasmid pUC-Apr-HA2-HA3 (Fig. S1B) was constructed. The second loxP site which was incorporated into oligo was recombined into pUC19 plasmid along with apramycin resistance gene (*aac(3)IV* gene) to generate pUC-Apr. Two ~1 kb homology arms corresponding to the 3′ end of the gene clusters of interest (HA2 and HA3, respectively) can then be cloned into pUC-Apr sequentially to obtain pUC-Apr-HA2-HA3. The HA2-Apr-loxP-HA3 fragment released from pUC-Apr-HA2-HA3 by restriction digestion is then electroporated into bacterium and integrated into the chromosome by a double-crossover event. At this point, the second loxP site has been introduced at the 3′ end of the gene cluster of interest.

Subsequently, electroporate the Cre recombinase expression plasmid pGB-hyg-Ptet-cre (Fig. S1C) which consists of *cre* gene placed under the control of tetracycline inducible promoter (Ptet), broad-host-range RK2 replicon (*oriV* and *trfA* gene), ColE1 origin, hygromycin and ampicillin resistance genes into bacterium. Induction of Cre recombinase expression with anhydrotetracycline could result in excising DNA fragment between two directly repeated loxP sites and producing a circular DNA molecule which consists of gene cluster of interest, BAC backbone, ampicillin, kanamycin and apramycin resistance genes. Subsequently, isolate plasmid DNA from induced culture and then electroporate into *E. coli*, select recombinants on ampicillin plus apramycin LB plate. Thus far, the complete gene cluster has been successfully cloned into pBeloBAC11.

### Application of “Cre/loxP plus BAC” strategy to clone type III secretion system (T3SS) gene cluster from TT01

**Figure 2:**
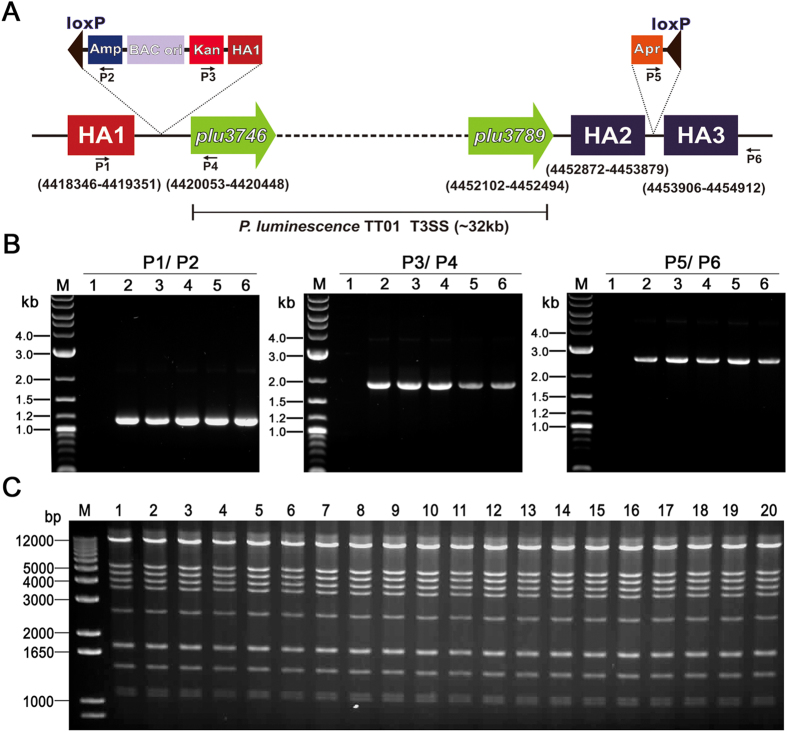
The application of “Cre/loxP plus BAC” strategy in *P. luminescens* TT01. (**A**) The schematic diagram showing the position of T3SS gene cluster in chromosome and the locations of inserted cassettes and checking primers (P1, P2, P3, P4, P5, P6). Numerals indicate nucleotide position of three homology arms (HA1, HA2 and HA3) and first ORF (*plu3746*) and last ORF (*plu3789*) of T3SS gene cluster. (**B**) Identification of integrants by Colony PCR with primer pairs P1/P2 (left panel), P3/P4 (middle panel) and P5/P6 (right panel) respectively. M, DNA marker; 1, negative control; 2 ~ 6, five different integrants picked from antibiotic plates. (**C**) Restriction analysis of pBeloBAC-pluT3SS. Twenty colonies were randomly picked and analyzed by DNA isolation and EcoRI digestion. M, DNA marker; 1 ~ 20, EcoRI digests of pBeloBAC-pluT3SS.

*P. luminescence* is an entomopathogenic bacterium belonging to the Enterobacteriaceae^27^. Several megasynthetase gene clusters of TT01 have previously been cloned by using RecET-mediated LLHR in our lab^18^. Genomic analysis of TT01 predicted the presence on the chromosome of gene cluster (*plu3746~plu3789*, 32 kb in size, Fig. 2A) encoding a T3SS, which has been proven to play a key role in determining the preferential extracellular location of *P. luminescence* in the haematopoietic organ of *Locusta*^28^,^29^,^30^. We firstly validated “Cre/loxP plus BAC” strategy for direct cloning of T3SS gene cluster from TT01.

The positions of homology arms (HA1, HA2 and HA3) on chromosome of TT01 were indicated in Fig. 2A. The size of all three homology arms was around 1 kb, which is sufficient for integration of DNA fragment into chromosome by RecA-mediated homologous recombination. In both integration events (single-crossover for the first loxP site and BAC integration and double-crossover for the second loxP integration, respectively), several tens or even hundreds of transformants appeared on LB plate containing appropriate antibiotic (kanamycin for single-crossover event, apramycin for double-crossover event) for every single electroporation. Three primer pairs (P1/P2, P3/P4 and P5/P6) were designed for identifying transformants by colony PCR, the results of which demonstrated the precise integration of the targeting DNA fragments at targeted loci (Fig. 2B). DNA sequencing of PCR products further confirmed the accuracy of integration events.

Induction of the saturated overnight culture of TT01 recombinants with 1 μg ml^−1^ anhydrotetracycline for 1 h rendered 53 out of 100 colonies sensitive to apramycin. Neither increased anhydrotetracycline concentration (2 μg ml^−1^, 4 μg ml^−1^ or 6 μg ml^−1^) or prolonged induction time (2 h, 4 h, 6 h) improved the excision efficiency. Shifting the induction temperature from 30 °C to 37 °C did not show significant difference in excision efficiency. As the optimal growth temperature of TT01 is close to 30 °C, we grew the saturated overnight culture of TT01 recombinants at 30 °C for 1 h after addition of anhydrotetracycline to a final concentration of 1 μg ml^−1^ to produce highest amount of excised circular DNA.

Isolation DNA from induced culture followed by electroporation into *E. coli* was performed by using the alkaline lysis method, which would denature chromosome DNA. Normally, 50 ~ 100 transformants would appear on ampicillin plus apramycin LB plate for every single electroporation. Twenty colonies were randomly picked and analyzed by DNA isolation and EcoRI digestion. The restriction pattern fit quite well with the expected (Fig. 2C), indicating the successful cloning of T3SS encoding gene cluster from TT01. Sequencing the flanking regions of loxP site resident in pBeloBAC-pluT3SS confirmed the precise excision catalyzed by Cre recombinase. To explore the possibility of unexpected rearrangement of pBeloBAC-pluT3SS, three colonies were subcultured independently for ten times in LB medium supplemented with ampicillin and apramycin followed by DNA isolation and restriction analysis, the result of which revealed that pBeloBAC-pluT3SS could be stably maintained in *E. coli* GB2005.

### Cloning of large DNA fragment from C58 by using “Cre/loxP plus BAC” strategy

The successful application of “Cre/loxP plus BAC” strategy in TT01 encouraged us to widen its usage in other bacteria, and also to direct clone DNA fragment of larger size. We then tested the validity of this strategy in C58 by attempting to direct clone a 78 kb DNA fragment containing the siderophore biosynthetic gene cluster (~45 kb)^31^.

**Figure 3:**
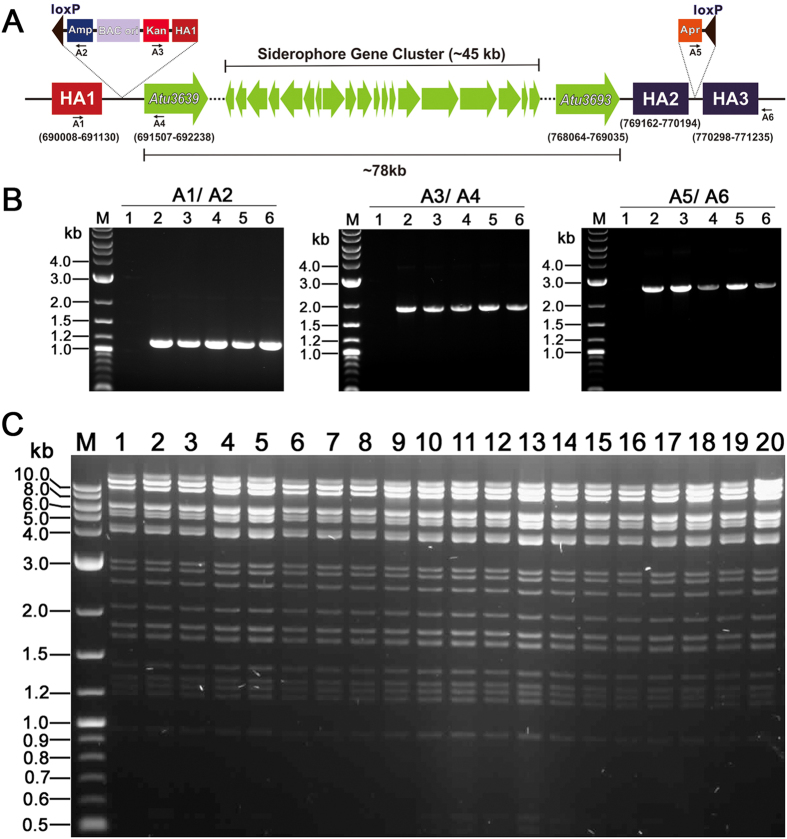
The application of “Cre/loxP plus BAC” strategy in *A. tumefaciens* C58. (**A**) The schematic diagram showing the position of cloned DNA fragment in chromosome and the locations of inserted cassettes and checking primers (A1, A2, A3, A4, A5, A6). Numerals indicate nucleotide position of three homology arms (HA1, HA2 and HA3) and first ORF (*Atu3639*) and last ORF (*Atu3693*) of cloned DNA fragment. (**B**) Identification of integrants by Colony PCR with primer pairs A1/A2 (left panel), A3/A4 (middle panel) and A5/A6 (right panel) respectively. M, DNA marker; 1, negative control; 2 ~ 6, five different integrants picked from antibiotic plates. (**C**) Restriction analysis of pBeloBAC-AgS. Twenty colonies were randomly picked and analyzed by DNA isolation and XhoI digestion. M, DNA marker; 1 ~ 20, XhoI digests of pBeloBAC-AgS.

Similarly, three 1 kb homology arms (HA1, HA2 and HA3) were generated and their positions on chromosome of C58 were indicated in Fig. 3A. Probably due to the lower electroporation efficiency in C58, the number of transformants obtained after each integration event (20 ~ 60) was less than that in TT01. But anyhow it was efficient enough to get positive transformants. Identification of transformants by colony PCR with three primer pairs (A1/A2, A3/A4 and A5/A6) (Fig. 3B) and subsequent DNA sequencing demonstrated the accuracy of integration events.

The optimum induction parameters for Cre recombinase in C58 were basically similar to that in TT01 except that 2 μg ml^−1^ anhydrotetracycline was more efficient than 1 μg ml^−1^ anhydrotetracycline. The maximum excision efficiency of Cre recombinase obtained in this case, excision of 78 kb DNA fragment from the genome of C58, was 23%. Electroporation of DNA isolated from induced culture into GB2005 produced 20 ~ 50 transformants on ampicillin plus apramycin LB plate. Transformants were picked and analyzed by DNA isolation and XhoI digestion (Fig. 3C), the result of which indicated that 78 kb DNA fragment containing the siderophore biosynthetic gene cluster has been successfully cloned into pBeloBAC11. Sequencing the flanking regions of loxP site resident in pBeloBAC-AgS confirmed the precise excision catalyzed by Cre recombinase. Stability test experiment demonstrated that pBeloBAC-AgS could be stably maintained in GB2005.

**Figure 4:**
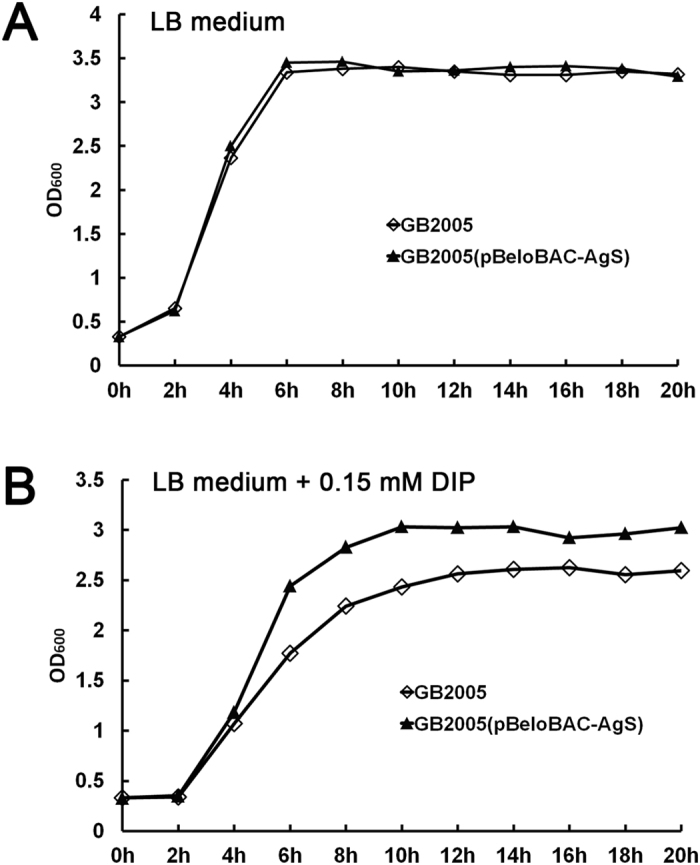
The growth curves of GB2005 and GB2005(pBeloBAC-AgS) in LB medium (**A**) and LB medium supplemented with 0.15 mM DIP (**B**).

In order to confirm the integrity of the cloned DNA fragment, the heterologous synthesis of C58 siderophore in *E. coli* GB2005 was detected by using high-performance liquid chromatography (HPLC). Unfortunately, we failed to directly detect C58 sideropore synthesis in GB2005. This may due to the low yield of C58 siderophore synthesis in the heterologous host. Actually, due to the trace amount of siderophore in C58, conflicting reports have been published concerning the ability of this strain to synthesize siderophores. Leong & Neilands found that C58 synthesized a siderophore^32^, but Penyalver *et al*. later reported that no detectable siderophores were produced by this strain^33^. As siderophore was known to be involved in chelating iron and allowing bacteria to proliferate in an iron-poor environment^31^, the ability of GB2005 recombinants harboring siderophore gene cluster from C58 to grow in liquid medium under iron-limiting conditions was examined. We grew GB2005 and GB2005(pBeloBAC-AgS) in LB liquid culture with or without 0.15 mM DIP and measured OD_600_ values every 2 h. GB2005(pBeloBAC-AgS) and GB2005 showed similar growth rates in LB medium without DIP (Fig. 4A) during the whole growth curve. When 0.15 mM DIP was supplemented in LB medium, the growth of both strains has been inhibited. However, GB2005(pBeloBAC-AgS) grew significantly better than GB2005 in the presence of 0.15 mM DIP (Fig. 4B), indicating that harboring siderophore gene cluster from C58 rendered GB2005 more resistance to iron starvation.

## Discussion

With the aim of direct cloning of gene clusters from microorganisms, we developed an alternative strategy termed as “Cre/loxP plus BAC”, which is based on Cre/loxP system and requires two steps of RecA-mediated homologous recombination. Recently, another site-specific recombinase system, φBT1 *attP-attB-int* which belongs to serine recombinase family has been adopted dedicatedly for *Streptomyces* genome engineering and cloning of antibiotic gene clusters^11^. However, we envision several advantages of “Cre/loxP plus BAC” strategy.

Firstly, the validity of this strategy is not restricted to a somewhat narrow range of organisms. Although we have only demonstrated its utility in *P. luminescens* and *A. tumefaciens*, both of which are Gram-negative bacteria, it is still imaginable that this strategy could be widely applied to other organisms, including *Streptomyces* genus, a treasure trove of nature porduct biosynthetic clusters. We made this comment based on two facts. (i) The Cre/loxP system has successfully used for genomic engineering in a wide range of organisms, and (ii) RecA is a ubiquitous protein present in all organisms ranging from bacteria to humans and RecA-mediated homologous recombination is essential for maintaining genomic integrity and for generating genetic diversity^34^. Secondly, the ability of this strategy to clone large DNA fragment will not disappoint natural product researchers. We demonstrated here that “Cre/loxP plus BAC” strategy could be used to efficiently direct clone DNA fragment in size up to 78 kb from C58. Actually, Cre/loxP system has been applied to delete much larger regions in both prokaryotic and eukaryotic organisms, which has been demonstrated by a couple of examples: (i) deletion of 1.5 Mb genomic region in genome of *Streptomyces avermitilis*^35^, and (ii) deletion of 4 Mb genomic region in genome of mouse embryonic stem (ES) cells^36^. The excellent excision activity of Cre/loxP coupled with the large DNA carry capacity of BAC origin would allow us to anticipate directly cloning much larger genomic DNA fragments. Thirdly, the choice of *E. coli* as host for cloning and maintenance the heterologous gene clusters provides the convenience of DNA modification. Mobile genetic elements (*mob, oriT*), resistance genes, as well as inducible promoters can be easily recombined into the constructs to meet the needs of expression in a heterologous host. In some cases, genes responsible for natural products biosynthesis are located remotely from the gene cluster and needed to be assembled into the gene cluster for heterologous expression. Furthermore, gene duplication, recombination, point mutation and module-skipping have often been used to modify gene clusters to comprehend the molecular mechanism of megasythnases and to produce new natural products^37^. With the aid of various recombineering systems and strategies established in our lab, the above mentioned DNA modifications can be easily achieved.

Concerning the practical application of “Cre/loxP plus BAC” strategy, several points should be noted. Firstly, the introduction of selection markers at both ends of targeted DNA region, as well as their location between two loxP sites contributes efficiently to screening of positive transformants. As BAC is integrated into the chromosome via single-crossover event, it can be efficiently excised by a reversal of the integration reaction between two copies of HA1 homology arm. Without the selective pressure of apramycin, several hundreds of colonies appeared on ampicillin and kanamycin LB plate for a single electroporation. However, more than ninety percent were false positive. That means when apramycin was not included in LB plate, the majority of colonies bear pBeloBAC-HA1 but without carrying the gene cluster of interest. This was obviously due to the fact that smaller plasmid (pBeloBAC-HA1) transformed at higher efficiencies than larger plasmid (pBeloBAC-pluT3SS, pBeloBAC-AgS). Secondly, the excision efficiency of Cre recombinase is positively correlated with the number of transformants. The optimum induction conditions for Cre recombinase activity obtained in *P. luminescens* and *A. tumefaciens* may not be applicable to other bacterial species. Taking the inducer concentration for example, the optimum concentration of anhydrotetracycline in C58 (2 μg ml^−1^) was higher than that in TT01 (1 μg ml^−1^). We hypothesize this because that a *tet(A)* class of tetracycline resistance gene present in the chromosome of C58^38^, the product of which could probably export anhydrotetracycline out of cell, resulting in reduced intercellular concentration of anhydrotetracycline. Therefore, it is necessary to determine the optimum conditions for Cre recombinase activity when applied to other bacterial species. Surely, genetic engineering could be also used to improve the expression of Cre recombinase, for instance, codon-optimized *cre* recombinase gene could be synthesized^39^,^40^, Ptet promoter could be replaced by more efficient inducible promoters.

In this study, we described the development of “Cre/loxP plus BAC” strategy and its application in two bacterial species. Owing to the merits described above, we believe that this strategy could be widely used for direct cloning of large DNA fragment from genome.

## Methods

### Strains, plasmids and reagents

Strains and plasmids used in this study are listed in Table S1. Recombineering-proficient *E. coli* strain GB05-dir which is chromosomally integrated *recE* and *recT* genes was used for plasmids construction^41^. *E. coli* GB2005 was used as cloning host. All *E. coli* strains were cultured in Luria-Bertani (LB) broth at 37 °C. *P. luminescens* subsp. *laumondii* TT01 and *A. tumefaciens* C58 (hereafter C58) were grown with aeration in LB broth at 30 °C.

The plasmid pGB-hyg-Ptet-gbaA which consists of tetracycline inducible promoter (Ptet), broad-host-range RK2 replicon (*oriV* and *trfA* gene), ColE1 origin, hygromycin and ampicillin resistance genes was used to construct Cre expression plasmid by replacing *redα-redβ-redγ-recA* operon with *cre* gene. The resultant plasmid was designated as pGB-hyg-Ptet-cre.

The widely used BAC vector, pBeloBAC11 was used to carry and stably maintain large DNA fragments containing the gene clusters of interest. The chloramphenicol resistance gene of pBeloBAC11 was replaced by kanamycin resistance gene. One loxP site was incorporated into the forward oligo for amplifying ampicillin resistance gene and introduced into pBeloBAC11 simultaneously. There exist EcoRІ, SacІ, BamHІ and HindIII unique restriction sites between kanamycin resistance gene and loxP site, which can be used to linearize the modified pBeloBAC11. DNA fragment containing homology arm (HA1) corresponding to the 5′ end of the gene clusters of interest was recombined into the modified pBeloBAC11 to generate pBeloBAC-HA1.

Another loxP site was incorporated into the reverse oligo for amplifying apramycin resistance gene and introduced into pUC19 plasmid simultaneously to generate pUC-Apr. Two DNA fragments containing homology arms (HA2 and HA3) corresponding to the 3′ end of the gene clusters of interest were recombined to pUC-Apr sequentially to obtain pUC-Apr-HA2-HA3. The HA2-Apr-loxP-HA3 fragment could be released from pUC-Apr-HA2-HA3 by restriction digestion.

Restriction enzymes, Phusion polymerase and DNA marker were purchased from New England Biolabs. Anhydrotetracycline and antibiotics were purchased from Sigma-Aldrich. Appropriate antibiotics were added at the following concentrations: for *E. coli* strains, ampicillin at 50 μg ml^−1^ (liquid) or 100 μg ml^−1^ (solid), hygromycin at 25 μg ml^−1^ (liquid) or 30 μg ml^−1^ (solid), apramycin at 10 μg ml^−1^ (liquid) or 20 μg ml^−1^ (solid), kanamycin at 15 μg ml^−1^ (both liquid and solid); for TT01, ampicillin at 100 μg ml^−1^ (both liquid and solid), kanamycin at 15 μg ml^−1^ (both liquid and solid), apramycin at 25 μg ml^−1^ (both liquid and solid); for C58, hygromycin at 50 μg ml^−1^ (both liquid and solid), apramycin at 50 μg ml^−1^ (both liquid and solid), kanamycin at 50 μg ml^−1^ (both liquid and solid).

### Electroporation procedures

For *E. coli*, electroporation was performed as previously described^41^. For TT01 and C58, 60 μl of overnight culture was transferred into 1.4 ml fresh LB medium supplemented with appropriate antibiotics if necessary and shaken at 30 °C for 3 h (for TT01) or 4 h (for C58) until the OD_600nm_ was around 0.6 ~ 0.8. Cells were harvested by centrifugation at 4 °C, 10000 rpm for 30 s. The supernatant was discarded, and the cell pellet was resuspended in 1 ml ice-cold GH buffer (10% glycerol, 2-mM HEPES, pH 6.5) and centrifuged. The ice-cold GH buffer washing procedure repeated once. Cells were finally resuspended in 30 μl ice-cold GH buffer and DNA was added. The mixture of electro-competent cells and DNA was transferred into an ice-cold 1 mm cuvette and shocked once using Eppendorf electroporator 2510 (Eppendorf, Germany) set at 1350 v. After electroporation, 1 ml LB medium was added into the cuvette and the cells were recovered at 30 °C for 2 hours with shaking and then plated on LB plates appropriate antibiotics. This was followed by incubation at 30 °C for 24 h (for TT01) or 48 h (for C58). Colony PCR was used to check recombinants and purified PCR products were subjected to sequencing to ensure the validity of every integration event.

### Recombineering in *E. coli* GB05-dir

Recombineering was performed according to the method established by our group^41^. Briefly, 40 μl of overnight culture of GB05-dir was transferred into 1.4 ml fresh LB medium and grown at 30 °C for 2 h. Then, 20 μl of 10% L-arabinose was added into the culture to induce the expression of Red recombinases and cells were shifted to 37 °C and shaken for another 40 min. Cells were harvested by centrifugation, washed twice with ice-cold sterile water, and finally resuspended in 50 μl ice-cold sterile water. Recombineering proficient competent cells mixed with DNA were transferred to an ice-cold 1 mm cuvette and shocked once using Eppendorf electroporator 2510 (Eppendorf, Germany) set at 1250 v. After electroporation, 1 ml LB medium was added into the cuvette and cells were recovered at 37 °C for 1 h with shaking and plated on LB plates supplemented with appropriate antibiotics, followed by an incubation at 37 °C for 24 h.

### Induction of Cre recombinase expression, DNA isolation and electroporation

TT01 or C58 recombinant strains which contain two loxP sites flanking gene cluster of interest and harbor Cre expression plasmid pGB-hyg-Ptet-cre were inoculated into 2 ml LB medium supplemented with appropriate antibiotics and shaken overnight at 30 °C. Different amounts of anhydrotetracycline solution (final concentrations of 1, 2, 4, 6, 8 μg ml^−1^ respectively) were added into the saturated overnight cultures and grow cells at 30 °C for another 1 h, 2 h, 4 h or 6 h to induce the expression of Cre recombinase to mediate excision between two parallel loxP sites.

After induction, an aliquot (50 μl) was spread onto LB agar plate and incubated at 30 °C. Single colonies appeared on LB plate were picked and screened for ampicillin and apramycin resistance. The excision efficiency was calculated by dividing the number of total colonies by the number of ampicillin and apramycin sensitive colonies. The rest culture was subjected to DNA isolation by using the alkaline lysis method. Isolated DNAs were immediately electroporated into GB2005. Transformants appeared on LB plates supplemented with ampicillin and apramycin were counted and subjected to DNA isolation and restriction analysis.

### Quantitative growth curve

The growth curve of *E. coli* GB2005 recombinant strain GB2005(pBeloBAC-AgS) containing siderophore gene cluster from C58 was measured and compared to that of GB2005 under both normal and Fe-depleted conditions. An overnight culture of GB2005 or GB2005(pBeloBAC-AgS) was diluted 1:50 into fresh LB medium supplemented with or without 0.15 mM 2, 2′-dipyridyl (DIP) and shaken at 37 °C for 24 h. A 1 ml of aliquot was taken every 2 h and OD_600nm_ was measured.

### Plasmid stability test

GB2005(pBeloBAC-pluT3SS) or GB2005(pBeloBAC-AgS) was grown overnight at 37 °C in LB supplemented with ampicillin plus apramycin. The overnight culture was 100 times diluted with fresh LB and incubated at 37 °C with shaking and then subcultured 20 times (approximately 240 generations) at the same conditions. At subculturings, diluted cells were plated on LB agar plates without antibiotic, and single colonies were picked and subjected to restriction analysis.

## Additional Information

**How to cite this article**: Hu, S. *et al*. “Cre/loxP plus BAC”: a strategy for direct cloning of large DNA fragment and its applications in *Photorhabdus luminescens* and *Agrobacterium tumefaciens*. *Sci. Rep*. **6**, 29087; doi: 10.1038/srep29087 (2016).

## Supplementary Material

Supplementary Information
