# Supplementary material for: “Cre/loxP plus BAC”: a strategy for direct cloning of large DNA fragment and its applications in Photorhabdus luminescens and Agrobacterium tumefaciens
Source: Sci Rep. 2016 Jul 1;6:29087. doi: 10.1038/srep29087 (PMC4929569; doi:10.1038/srep29087)
Supplement: Supplementary Information [file srep29087-s1.doc]

**“Cre/loxP plus BAC”: a strategy for direct cloning of large DNA fragment and its applications in *Photorhabdus luminescens* and *Agrobacterium tumefaciens***

Shengbiao Hu^1, 3^, Zhengqiang Liu^1^, Xu Zhang^1^, Guoyong Zhang^1^, Yali Xie^1^, Xuezhi Ding^1^, Xiangtao Mo^1^, A. Francis Stewart^3^, Jun Fu^2, 3^, Youming Zhang^1, 2^ & Liqiu Xia^1^

*^1^Hunan Provincial Key Laboratory of Microbial Molecular Biology-State Key Laboratory Breeding Base of Microbial Molecular Biology, College of Life Science, Hunan Normal University, Changsha, 410081, People’s Republic of China.*

*^2^Shandong University-Helmholtz Institute of Biotechnology, State Key Laboratory of Microbial Technology, School of Life Science, Shandong University, Shanda Nanlu 27, Jinan, 250100, People’s Republic of China.*

*^3^Department of Genomics, Dresden University of Technology, BioInnovations-Zentrum, Tatzberg 47-51, Dresden, 01307, Germany.*

*Correspondence and requests for materials should be addressed to L.Q.X (email: xialq@hunnu.edu.cn) or J.F. (email: fujun@sdu.edu.cn) or Y.M.Z. (email: zhangyouming@sdu.edu.cn)*

**Supplementary materials**


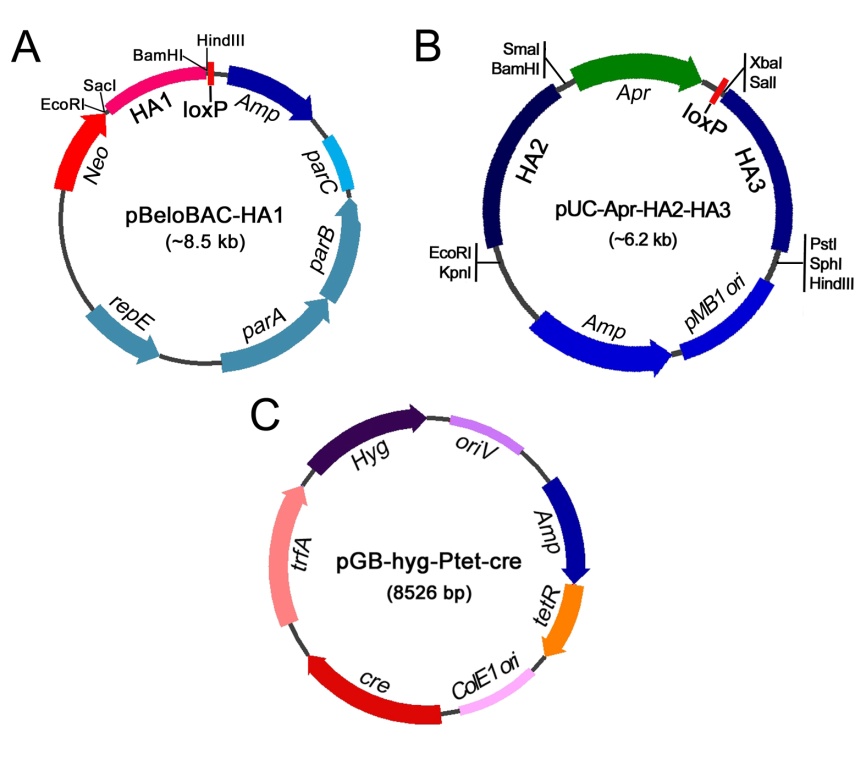


**Fig. S1 Profiles of plasmids constructed in this study. (A)** pBeloBAC-HA1 used for integrating the first loxP site and BAC backbone at the 5’ end of the gene clusters of interest. **(B)** pUC-Apr-HA2-HA3 used for integrating the second loxP site at the 3’ end of the gene clusters of interest. **(C)** Cre recombinase expression plasmid pGB-hyg-Ptet-cre, which consists of *cre* gene placed under the control of tetracycline inducible promoter (Ptet), broad-host-range RK2 replicon (*oriV* and *trfA* gene), ColE1 origin, hygromycin and ampicillin resistance genes.

**Table S1 Strains and plasmids**

| Strains and Plasmids | Characteristics | References or sources |
| --- | --- | --- |
| *Escherichia coli* |  |  |
| GB2005 | F-*mcr*A ∆(*mrr*-*hsd*RMS-*mcr*BC) *φ*80*lac*Z∆M15 ∆*lac*X74 *rec*A1 *end*A1 *ara*D139 ∆(*ara, leu*)7697 *gal*U *gal*K λ*rpsL nup*G*fhu*A::IS2 *rec*ET *redα*, phage T1-resistent | 1 |
| GB05-dir | Recombineering-proficient strain, chromosomally integrated *recE* and *recT* genes in GB2005 | 2 |
| GB2005(pBeloBAC-AgS) | GB2005 harboring plasmid pBeloBAC-AgS | This study |
| *P. luminescens* TT01 | Wild-type strain | DSM 15139 |
| *A. tumefaciens* C58 | Wild-type strain containing the nopaline-type Ti plasmid pTiC58 | ATCC 33970 |
| Plasmids |  |  |
| pBeloBAC11 | Cloning vector, Cm^r^, genBank accession no. U51113 | 3 |
| pBeloBAC-HA1 | pBeloBAC11 containing a loxP site, Amp^r^ and Kan^r^ | This study |
| pBeloBAC-pluT3SS | pBeloBAC11 containing T3SS gene cluster from *P. luminescens* TT01 | This study |
| pBeloBAC-AgS | pBeloBAC11 containing siderophore gene cluster from *A. tumefaciens* C58 | This study |
| pUC19 | Cloning vector, Amp^r^ | Lab store |
| pUC-Apr-HA2-HA3 | pUC19 containing a loxP site and apramycin resistance gene, Amp^r^ and Apr^r^ | This study |
| pGB-hyg-Ptet-gbaA | Recombineering expression plasmid replicable in *A. tumefaciens*, containing *redα/redβ/redλ/recA* genes, Amp^r^ and Hyg^r^ | 4 |
| pGB-hyg-Ptet-cre | Cre recombinase expression plasmid, containing *cre* gene placed under the control of tetracycline inducible promoter (Ptet) | This study |

**Table S2 Oligos**

| Name | Sequence* | Description |
| --- | --- | --- |
| Kan-F | GCATATCCACTCAGTTCCACATTTCCATATAAAGGCCAAGGCATTTATTCTCACGCTGCCGCAAGCACTC | amplification of kanamycin resistance gene for the replacement of chloramphenicol resistance gene resident in pBeloBAC11 |
| Kan-R | GCCGGCACGTTAACCGGGCTGCATCCGATGCAAGTGTGTCGCTGTCGACGTCAGAAGAACTCGTCAAGAAG |
| Cre-F | GAGAAAAGTGAAATGAATAGTTCGACAAAAATCTAGCAGGAGGAATTCATATGTCCAATTTACTGACCGTAC | amplification of Cre recombinase encoding gene |
| Cre-R | CATGCCGACACGTTCAGCCAGCTTCCCAGCCAGCGTTGCGAGTGCAGTACCTAATCGCCATCTTCCAGCAGG |
| Amp-F | CCGATTCGCAGCGCATCGCCTTCTATCGCCTTCTTGACGAGTTCTTCTGA*GAATTCGAGCTCGGATCCAAGCTT*ATAACTTCGTATAGCATACATTATACGAAGTTATTAGACGTCAGGTGGCACTTTTC | amplification of ampicillin resistance gene |
| Amp-R | GCCGGCACGTTAACCGGGCTGCATCCGATGCAAGTGTGTCGCTGTCGACGTTCAAAAAAAAGCCCGCTC |
| Apr-F | GACGTTGTAAAACGACGGCCAGTGAATTCGAGCTCGGTACCCGGGGATCCACGCTCAGTGGAACGAGGTTC | amplification of apramycin resistance gene |
| Apr-R | GCTATGACCATGATTACGCCAAGCTTGCATGCCTGCAGGTCGACTCTAGAATAACTTCGTATAATGTATGCTATACGAAGTTATTCAGCCAATCGACTGGCGAGC |
| PHA1-F | ATCGCCTTCTTGACGAGTTCTTCTGAGAATTCGAGCTCGGATCCAAGCTTGCCGCCATTAGGCATATTC | amplification of homology arm 1 of *P. luminescens* TT01 chromosome |
| PHA1-R | TGCCACCTGACGTCTAATAACTTCGTATAATGTATGCTATACGAAGTTATAGAGCGATATCCTAATGCG |
| PHA2-F | TTCCCAGTCACGACGTTGTAAAACGACGGCCAGTGAATTCGAGCTCGGTACAGTCGCTAATGAAACACAG | amplification of homology arm 2 of *P. luminescens* TT01 chromosome |
| PHA2-R | GCTGATGGAGCTGCACATGAACCTCGTTCCACTGAGCGTGGATCCCCGGGTATTCGCTACCGCGGTGGGTC |
| PHA3-F | CCAGTCGATTGGCTGAATAACTTCGTATAGCATACATTATACGAAGTTATGTAAGACACCACACCACAG | amplification of homology arm 3 of *P. luminescens* TT01 chromosome |
| PHA3-R | GCTATGACCATGATTACGCCAAGCTTGCATGCCTGCAGGTCGACTCTAGATTGGTGCAGACATTATGCCCAC |
| AHA1-F | ATCGCCTTCTTGACGAGTTCTTCTGAGAATTCGAGCTCGGATCCAAGCTTTCTCAAAAGCCTCACGAAGC | amplification of homology arm 1 of *A. tumefaciens* C58 chromosome |
| AHA1-R | TGCCACCTGACGTCTAATAACTTCGTATAATGTATGCTATACGAAGTTATTGTACCGGCGAACTGGTTCC |
| AHA2-F | TTCCCAGTCACGACGTTGTAAAACGACGGCCAGTGAATTCGAGCTCGGTATACTCGACAAAAATCCGCAC | amplification of homology arm 2 of *A. tumefaciens* C58 chromosome |
| AHA2-R | GCTGATGGAGCTGCACATGAACCTCGTTCCACTGAGCGTGGATCCCCGGGCATTCATCTATGGCCTTGCCTTC |
| AHA3-F | CCAGTCGATTGGCTGAATAACTTCGTATAGCATACATTATACGAAGTTATGCCGGTCTGCGCATCAGCCATG | amplification of homology arm 3 of *A. tumefaciens* C58 chromosome |
| AHA3-R | GCTATGACCATGATTACGCCAAGCTTGCATGCCTGCAGGTCGACTCTAGAGGCGGAAAAAGGCTGTCATC |
| P1 | TGCCAAAGACATGGTTCTGC | checking primer pair in *P. luminescens* TT01 |
| P2 | GGTTAGCTCCTTCGGTCCTC |
| P3 | ACCGCTTCCTCGTGCTTTAC |
| P4 | TGTTAATGGCCTGTTTTTTC |
| P5 | AGGCCCTGCGTGCTGCGCTG |
| P6 | AGGCTACGGTATTAATTGAC |
| A1(=P1) | CCTTAAATCGTGTAACGGAC | checking primer pair in *A. tumefaciens* C58 |
| A2 | GGTTAGCTCCTTCGGTCCTC |
| A3(=P3) | ACCGCTTCCTCGTGCTTTAC |
| A4 | TTGTCATTCAACGTCTCTCC |
| A5(=P5) | AGGCCCTGCGTGCTGCGCTG |
| A6 | CGCCGGGTTACGGCGATCTG |

* Homology arms for recombineering were underlined. loxP sites were double underlined. Restriction sites were italic.

**Supplementary References**

1. Fu, J., Teucher, M., Anastassiadis, K., Skarnes, W. & Stewart, A.F. A Recombineering Pipeline to Make Conditional Targeting Constructs, Vol. 477. (Academic Press, Unit State; 2010).
2. Fu, J.*et al.*Full-length RecE enhances linear-linear homologous recombination and facilitates direct cloning for bioprospecting. *Nat. Biotechnol.***30**, 440-446 (2012).
3. Wang, K., Boysen, C., Shizuya, H., Simon, M. I., & Hood, L. (1997). Complete nucleotide sequence of two generations of a bacterial artificial chromosome cloning vector. *BioTechniques*, *23*(6), 992-994.
4. Hu, S., Fu, J., Huang, F., Ding, X., Stewart, A. F., Xia, L., & Zhang, Y. (2014). Genome engineering of Agrobacterium tumefaciens using the lambda Red recombination system. *Applied microbiology and biotechnology*, *98*(5), 2165-2172.
